# Expression and cellular trafficking of GP82 and GP90 glycoproteins during *Trypanosoma cruzi* metacyclogenesis

**DOI:** 10.1186/1756-3305-6-127

**Published:** 2013-05-01

**Authors:** Ethel Bayer-Santos, Narcisa Leal Cunha-e-Silva, Nobuko Yoshida, José Franco da Silveira

**Affiliations:** 1 Departamento de Microbiologia, Imunologia e Parasitologia, Universidade Federal de São Paulo, São Paulo, SP 04023-062, Brazil; 2 Instituto de Biofísica Carlos Chagas Filho, Universidade Federal do Rio de Janeiro, Rio de Janeiro, RJ, 21949-900, Brazil

**Keywords:** *Trypanosoma cruzi*, Metacyclogenesis, Surface proteins, Gene expression, Intracellular trafficking

## Abstract

**Background:**

The transformation of noninfective epimastigotes into infective metacyclic trypomastigotes (metacyclogenesis) is a fundamental step in the life cycle of *Trypanosoma cruzi*, comprising several morphological and biochemical changes. GP82 and GP90 are glycoproteins expressed at the surface of metacyclic trypomastigote, with opposite roles in mammalian cell invasion. GP82 is an adhesin that promotes cell invasion, while GP90 acts as a negative regulator of parasite internalization. Our understanding of the synthesis and intracellular trafficking of GP82 and GP90 during metacyclogenesis is still limited. Therefore, we decided to determine whether GP82 and GP90 are expressed only in fully differentiated metacyclic forms or they start to be expressed in intermediate forms undergoing differentiation.

**Methods:**

Parasite populations enriched in intermediate forms undergoing differentiation were analyzed by quantitative real-time PCR, Western blot, flow cytometry and immunofluorescence to assess GP82 and GP90 expression.

**Results:**

We found that GP82 and GP90 mRNAs and proteins are expressed in intermediate forms and reach higher levels in fully differentiated metacyclic forms. Surprisingly, GP82 and GP90 presented distinct cellular localizations in intermediate forms compared to metacyclic trypomastigotes. In intermediate forms, GP82 is localized in organelles at the posterior region and colocalizes with cruzipain, while GP90 is localized at the flagellar pocket region.

**Conclusions:**

This study discloses new aspects of protein expression and trafficking during *T. cruzi* differentiation by showing that the machinery involved in GP82 and GP90 gene expression starts to operate early in the differentiation process and that different secretion pathways are responsible for delivering these glycoproteins toward the cell surface.

## Background

The metacyclogenesis, a process that involves the transformation of noninfective epimastigotes into infective metacyclic trypomastigotes, is a fundamental step in the life cycle of the protozoan *Trypanosoma cruzi*, the etiological agent of Chagas disease. It occurs at the hindgut of the insect vector, where epimastigotes attach to the superficial cuticle layer of the gut epithelium prior to differentiation into metacyclic trypomastigotes [1-3]. Metacyclic forms detached from the hindgut wall are released in the insect’s feces during its blood meal and enter mammalian host cells through skin lesions. Another mode of transmission is the oral infection (reviewed in [4]), where whole triatomine insects or their feces containing metacyclic forms are potential sources of food contamination.

It is not clear how the differentiation process is triggered, but nutritional stress and adhesion to substrate are important requirements, with the involvement of free fatty acids, cyclic AMP and adenylate cyclase [5-8]. Several changes occur during metacyclogenesis, including nuclear structure modifications [9], chromatin remodeling and differential mRNA stability [10,11], which result in differential protein expression [12], changes in cell morphology [13], proliferation and infectivity. It has been demonstrated that the expression of some stage-specific genes precedes morphological changes during metacyclogenesis [11,14,15]. Additionally, changes in membrane lipids [16] and carbohydrate composition [17] were observed to precede morphological transformation.

The *trans*-sialidase family members, GP82 and GP90, are developmentally regulated proteins expressed in metacyclic trypomastigotes [18-21], in which their mRNA half-life is longer than in epimastigotes [22]. These molecules play distinct roles in parasite internalization: the cell invasion-promoting GP82 induces a transient increase in host cell intracellular Ca^2+^ concentration [23,24] and actin cytoskeleton disruption [25], leading to the recruitment of lysosomes to the site of entry [26], an event required for the biogenesis of parasitophorous vacuole and host cell invasion [27]. GP90 binds to mammalian cells without triggering Ca^2+^ signals and functions as a down regulator in cell invasion so that its expression is inversely correlated with the parasite’s capacity to invade mammalian cells [24,28].

Despite GP82 and GP90 have been known for many years, our understanding of their synthesis and intracellular trafficking during the metacyclogenesis is still limited. A previous attempt to clarify this issue gave contradictory results that were unable to be clarified due to technical limitations imposed by the *in vivo* system [29]. Therefore, here we decided to use reproducible axenic culture conditions [30] to study the metacyclogenesis and determine whether GP82 and GP90 are expressed only in fully differentiated metacyclic forms or they start to be expressed in intermediate forms undergoing differentiation. Isolated intermediate forms were analyzed by a combination of techniques, revealing that GP82 and GP90 mRNAs and proteins are already expressed. Unexpectedly, GP82 and GP90 presented distinct cellular localizations in intermediate forms, indicating that during morphological changes they follow different pathways toward the surface of metacyclic trypomastigotes.

## Methods

### Ethics statement

This study was carried out in accordance with recommendations in the Guide for Care and Use of Laboratory Animals of the National Institutes of Health. The protocol was approved by the Committee on Animal Experiment Ethics of Universidade Federal de São Paulo (Protocol Number: CEP09555-07).

### Parasites and *in vitro* metacyclogenesis

*T. cruzi* G strain [31] was maintained alternately in mice and in liver infusion tryptose (LIT) medium, containing 10% fetal bovine serum at 28°C. Metacyclogenesis was induced according to the procedure described by Contreras *et al.*[5]. Briefly, epimastigotes were grown to stationary phase, collected by centrifugation, washed once in triatomine artificial urine (TAU) medium (190 mM NaCl, 17 mM KCl, 2 mM MgCl_2_, 2 mM CaCl_2_, 8 mM sodium phosphate buffer, pH 6.0) and diluted to 5 × 10^8^ cells/mL in the same medium. After 2 h at 28°C, parasites were diluted 100 fold in TAU supplemented with 50 mM sodium glutamate, 10 mM L-proline, 2 mM sodium aspartate and 10 mM glucose (TAU3AAG), allowed to attach to cell culture flasks and maintained afterwards at 28°C. Attached parasites were collected 24 and 48 h later by removing the supernatant, washing the attached cells once with TAU, and vigorously shaking the parasites with TAU medium. Metacyclic trypomastigotes were obtained from culture supernatants from TAU3AAG and purified by anion-exchange chromatography using DEAE-cellulose as previously described [18,31]. Briefly, parasites were washed twice with phosphate-buffered saline containing 5.4% glucose (PSG) pH 8.0, followed by passage through a DEAE-cellulose column packed in a 20 mL plastic syringe and elution with PSG.

### RNA extraction, cDNA synthesis and quantitative real-time PCR

Real-time PCR was performed to assess the expression of GP82 and GP90 mRNAs during metacyclogenesis. Total RNA was isolated from 5 × 10^7^ parasites using Trizol reagent (Invitrogen) and treated with RNAse-free DNase (Invitrogen). Two micrograms of total RNA was used for cDNA synthesis using the ThermoScript Preamplification System according to the manufacturer’s instructions (Invitrogen). Quantitative real-time PCR was mainly conducted as described earlier [29]. Briefly, reactions were carried out with 12.5 μL of SYBR-Green PCR master mix (Applied Biosystems), 1.6 μL of cDNA and primers at a final concentration of 200 nM in a final volume of 20 μL. PCR was conducted in the ABI Prism 7500 (Applied Biosystems) and analyzed with ABI Prism 7500 SDS version 2.0 software. cDNA from exponentially growing epimastigotes were used as a control for comparison purposes. All quantifications were normalized to the housekeeping gene glyceraldehyde 3-phosphate dehydrogenase (GAPDH, GenBank: X52898). It is worth mentioning that RT-PCR primers specifically detected GP82 and GP90 family members and results shown in here are derived from a subset of members sharing high sequence similarity.

### Western blot

Total proteins were extracted from 2.5 × 10^6^ parasites with a solution containing Tris-HCl 62 mM, 10% glycerol, 2% SDS, 5% β-mercaptoethanol, 0.01% bromophenol blue and boiled prior to sample loading on 8% SDS-PAGE. After electrophoresis, proteins were transferred to nitrocellulose membranes, blocked with 5% non-fat milk for 1 h at room temperature (RT) and incubated for 1 h at RT with monoclonal antibody (mAb) 3F6 or 1G7 against GP82 and GP90, respectively [18]. Membranes were washed four times for 5 min each with phosphate-buffered saline (PBS) containing 0.05% Tween 20, incubated with anti-mouse IgG coupled to horseradish peroxidase (Sigma) for 1 h at RT, washed again in the same conditions and visualized by chemiluminescence.

### Flow cytometry

Live parasites (4 × 10^7^) were incubated for 30 min on ice with mAbs 3F6 or 1G7 diluted in 1% bovine serum albumin (BSA)/PBS. After washings in PBS, cells were fixed with 4% paraformaldehyde (PFA) in PBS for 15 min. The fixative was washed out and the parasites were incubated with Alexa Fluor 488-conjugated anti-mouse IgG diluted in 1% BSA/PBS for 1 h at RT. After two more washes, the fluorescence was determined on a FACSCalibur II cytometer (Becton Dickinson) and data analysis performed using CellQuest software. Assays with permeabilized parasites were carried out as follows: fixation with 4% PFA, washes in PBS, incubation with 50 mM ammonium chloride for 15 min, washes in PBS, treatment with 0.1% saponin in 1% BSA/PBS at RT for 30 min, washes in PBS, incubation with mAb 3F6 or 1G7 diluted in 1% BSA/PBS for 1 h at RT, washes in PBS, incubation with Alexa Fluor 488-conjugated antibody and final washes in PBS.

### Immunofluorescence

Parasites were fixed with 4% PFA, incubated with 50 mM ammonium chloride, washed with PBS and let attach to glass slides pretreated with 0.01% poly-L-lysine. Parasites were then blocked and permeabilized with a solution containing 0.5% saponin and 1% BSA for 1 h. Cells were incubated with mAbs 3F6 or 1G7 diluted in 1% BSA and 0.1% saponin for 1 h at RT or with rabbit polyclonal antibody anti-cruzipain provided by Dr. Ana Paula C. A. Lima, UFRJ, Brazil. The preparations were washed with PBS, incubated with anti-mouse Alexa Fluor 488 and anti-rabbit Alexa Fluor 568 (Invitrogen) diluted in 1% BSA and 0.1% saponin for 1 h, together with 10 μM of DAPI (4′,6′-diamidino-2-phenylindole; Molecular Probes) to visualize the nucleus and kinetoplast. Images were acquired on an Olympus BX51 fluorescence microscope coupled to an Olympus DP71 digital camera using Image-Pro 6.2 software. It is worth mentioning that mAb 3F6 and 1G7 are specific to a subset of GP82 and GP90 family members sharing high sequence similarity.

### Statistical analysis

Student’s *t*-test was used to determine the significance in real-time PCR experiments in which GP82 and GP90 mRNA levels of exponentially growing epimastigotes were compared to intermediate and metacyclic forms and in flow cytometry assays where the fluorescence intensity was compared between live and permeabilized parasites.

## Results

### Expression of GP82 and GP90 during metacyclogenesis

*In vitro* differentiation was carried out using TAU3AAG medium, which allows parasites to attach to cell culture flasks while undergoing differentiation. Parasite forms that are close to completing differentiation into metacyclic trypomastigotes detach from culture flasks and stay in the supernatant while other forms that are still differentiating remain attached to culture flasks [6]. Thus, TAU3AAG medium is a good choice for quantitative analysis as it allows isolation of intermediate forms undergoing differentiation, with a minor contamination of metacyclic forms.

**Figure 1:**
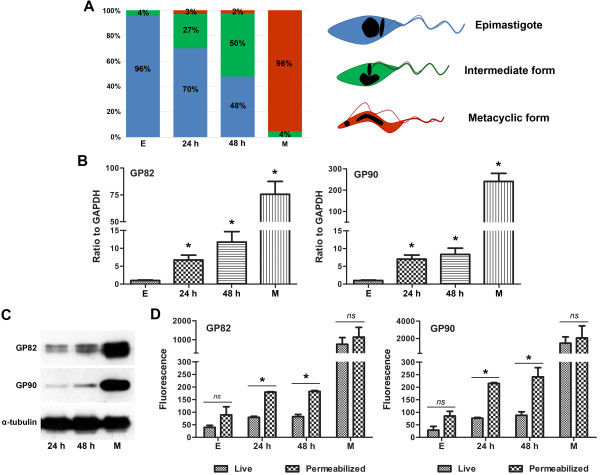
**Expression of GP82 and GP90 at different time-points during *T. cruzi *metacyclogenesis. **(**A**) Relative number of epimastigotes (blue), intermediate forms (green) and metacyclic trypomastigotes (red) in analyzed samples. Numbers are derived from three independent experiments where 200 cells were analyzed. A representative drawing of each parasite form is shown on the right. (**B**) Quantitative real-time PCR for determination of transcript levels. Means and standard deviations are derived from three independent experiments. The difference between epimastigotes and other forms was significant (P <0.05). (**C**) SDS-PAGE followed by Western blot showing the presence of GP82 and GP90 at 24 and 48 h. Total protein extract from 2.5 × 10^6 ^parasites were used and anti-α-tubulin mAb was used as loading control. Experiments shown were carried out three times with similar results. (**D**) Live or permeabilized parasites were reacted with mAb 3F6 or 1G7 and processed for flow cytometry analysis. Values represent fluorescence means and standard deviation of independent experiments that were normalized by the fluorescence of parasites incubated only with secondary antibody. The difference between live and permeabilized parasites was significant for 24 and 48 h samples (P <0.05) and not significant (*ns*) for epimastigotes and metacyclic forms.

The following developmental forms were analyzed: exponentially growing epimastigotes (E) obtained from LIT; intermediate forms attached to culture flasks 24 (24 h) and 48 h (48 h) after inoculum in TAU3AAG; and metacyclic trypomastigotes (M) obtained from TAU3AAG supernatant and purified by DEAE-cellulose. To distinguish *T. cruzi* developmental stages, DAPI staining and phase contrast were used to identify nucleus/kinetoplast and flagellum position. Based on parasite’s morphology, the relative number of epimastigotes, intermediate forms and metacyclic forms were estimated in each sample according to Ferreira *et al*. [13]. Epimastigote forms contain a spherical nucleus with a flagellum protruding from the anterior portion of the cell body near to the disk-shaped kinetoplast. Intermediate forms have a somewhat elongated nucleus with the kinetoplast varying in position relative to the nucleus, either anterior, at the middle or posterior. Metacyclic trypomastigotes have a fully elongated nucleus with a round kinetoplast at the posterior end of the parasite (see Figure 1A). Upon seeding in TAU3AAG medium for differentiation, the percentage of intermediate forms increased from the initial 4% in epimastigote cultures to 50% in samples collected at 48 h (Figure 1A), showing that this time point is enriched in intermediate forms with a minimal contamination of metacyclic trypomastigotes (2%). Such contamination did not interfere significantly in posterior analyses as according to flow cytometry results there is a shift in fluorescence intensity in the whole parasite population obtained at 24 h and 48 h compared to epimastigote sample, which would not be observed if the results obtained were driven by the 2-3% metacyclic forms (Additional file 1).

GP82 and GP90 mRNA are enriched in metacyclic trypomastigotes when compared to exponentially growing epimastigotes [22]. To investigate whether GP82 and GP90 mRNAs start to be stabilized in intermediate forms undergoing differentiation, total RNA was extracted and analyzed by quantitative real-time PCR. Results revealed that GP82 and GP90 mRNA levels are increased in parasites undergoing metacyclogenesis (24 and 48 h) when compared to exponentially growing epimastigotes (E) (Figure 1B). Intermediate forms collected at 48 h showed 11-fold and 8-fold increase in GP82 and GP90 mRNA levels when compared to epimastigotes, suggesting that mRNA stabilization occurs before the differentiation to metacyclic trypomastigotes (Figure 1B). Thus, GP82 and GP90 transcript levels increase during the differentiation process, reaching higher levels in metacyclic forms.

Total protein extracts of parasites attached to culture flasks at 24 and 48 h were analyzed by Western blot, revealing that GP82 and GP90 are already translated in intermediate forms (Figure 1C). To determine whether GP82 and GP90 were located in the plasma membrane surface or in intracellular compartments, live and permeabilized parasites were incubated with mAbs 3F6 and 1G7 and processed for flow cytometry (Figure 1D). The fluorescence signal was lower in live intermediate forms compared to permeabilized ones, indicating that GP82 and GP90 are mainly located in intracellular compartments. Statistical significant differences were observed between live and permeabilized parasites at 24 and 48 h, but no significant difference was observed for epimastigotes or metacyclic forms (Figure 1D). Together, these data show that the expression of GP82 and GP90 starts in intermediate stages during metacyclogenesis and reaches the highest level in fully differentiated metacyclic forms.

### Cellular localization of GP82 and GP90 during metacyclogenesis

**Figure 2:**
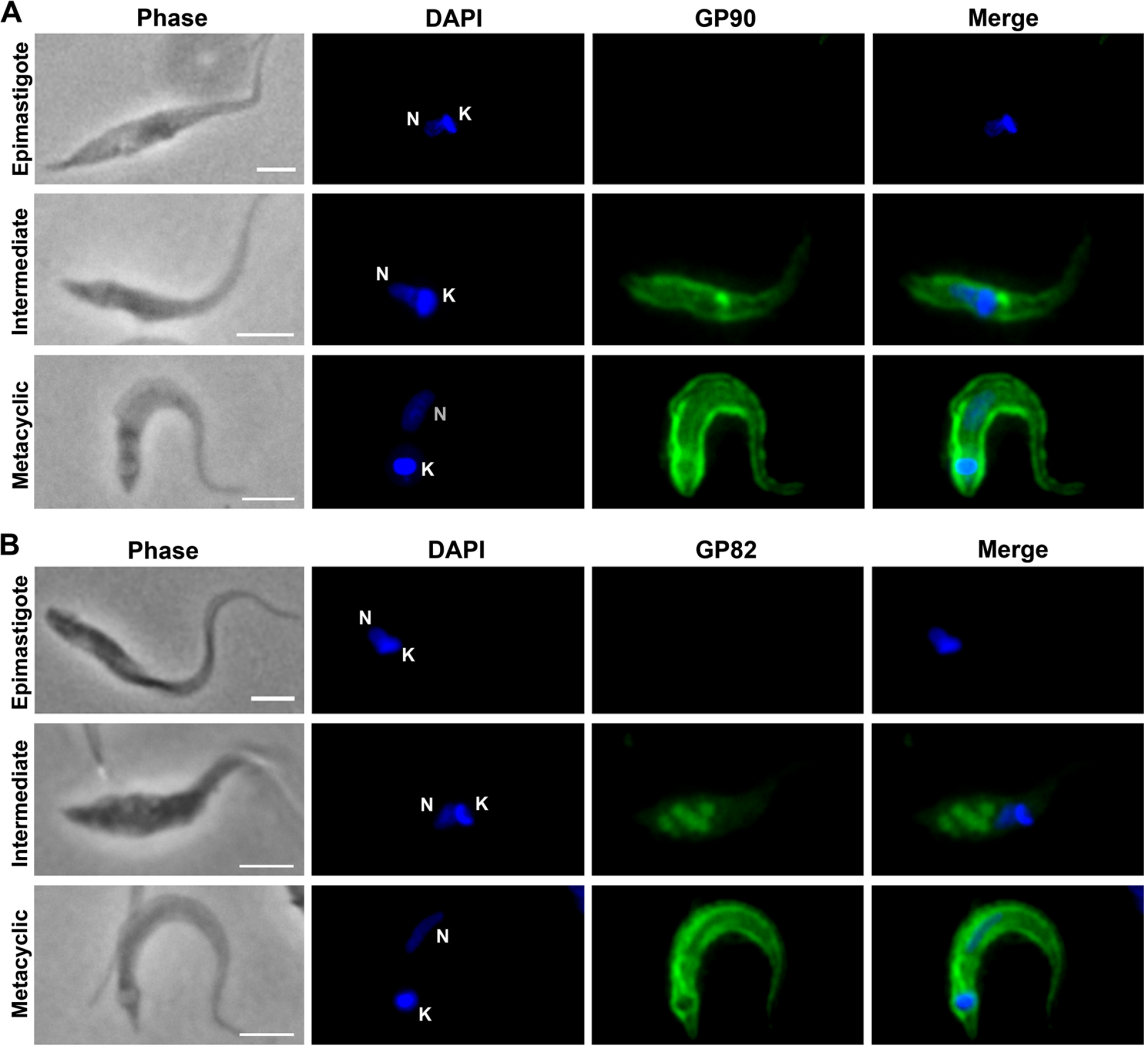
**Differential cellular localization of GP82 and GP90 in parasites undergoing metacyclogenesis. **Immunofluorescence was performed using exponentially growing epimastigotes, parasites attached to culture flasks at 48 h after nutritional stress and metacyclic trypomastigotes purified by DEAE-cellulose. Cells were fixed, permeabilized with 0.5% saponin, reacted with mAb 1G7 (**A**) or 3F6 (**B**) and incubated with secondary antibody Alexa Fluor 488. DAPI was used to stain nucleus (N) and kinetoplast (K). Scale bar = 10 μm.

In metacyclic trypomastigotes, GP82 and GP90 are found mainly at the plasma membrane [18]. Since both molecules are also expressed in intermediate forms, but their localizations appear to be predominantly intracellular (Figure 1D), indirect immunofluorescence was performed to assess GP82 and GP90 cellular localization. Distinct from exponentially growing epimastigotes that did not exhibit fluorescence signal and from metacyclic trypomastigotes that displayed a membrane labeling pattern for both GP82 and GP90; in intermediate forms, GP82 localized mainly in vesicular structures at the parasite posterior region, while GP90 localized at the plasma membrane and in the region close to the kinetoplast, where the Golgi complex and the flagellar pocket are located (Figure 2A and B). These labeling patterns, easily detected in parasites attached to culture flasks at 48 h, could be seen in a minor portion of the parasite population as soon as 12 h after nutritional stress (data not shown). In intermediate forms at 48 h, labeling with anti-GP82 mAb was detected in vesicular structures in 68 ± 6.5% of the cells, while anti-GP90 mAb labeled the region close to kinetoplast in 30 ± 1.5% of the cells (three independent experiments).

**Figure 3:**
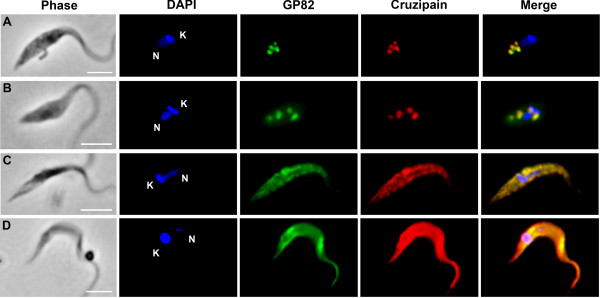
**Colocalization of GP82 with cruzipain during metacyclogenesis.** Immunofluorescence showing intermediate forms attached to culture flasks at 48 h (**A-B**) and late intermediate forms and fully differentiated metacyclic forms in culture supernatant (**C-D**) at 48 h after nutritional stress. Cells were fixed, permeabilized with 0.5% saponin and submitted to immunofluorescence using mAb 3F6 and rabbit polyclonal antibody against *T. cruzi *cruzipain. DAPI was used to stain nucleus (N) and kinetoplast (K). Scale bar = 10 μm.

GP82 and GP90 genes encode an N-terminal signal peptide and a C-terminal GPI anchor signal, which address these proteins to the endoplasmic reticulum (ER) and Golgi complex for addition of GPI anchor [32-34] and glycosylation [35,36]. In *T. cruzi*, the processes of protein secretion and trafficking to the plasma membrane occur mainly via the flagellar pocket, the only region where the parasite’s body is not covered by subpellicular microtubules [37]. Since GP82 and GP90 are glycoproteins located at the plasma membrane, they were expected to localize in the Golgi complex and flagellar pocket of intermediate forms, as observed for GP90 (Figure 2A). However, in contrast to GP90 localization, GP82 was detected in vesicular structures at the posterior region of intermediate forms (Figure 2B), indicating that the trafficking of these proteins occurs through distinct pathways. To investigate whether these vesicular structures correspond to late endosomes named reservosomes or lysosomes-related organelles (LROs), which are localized at *T. cruzi* posterior region [38,39], assays were performed to assess the colocalization with the cysteine proteinase cruzipain that is abundant in LROs [40]. Figure 3 shows GP82 colocalization with cruzipain at the posterior region of attached intermediate forms (Figure 3A), as well as in structures close to the kinetoplast in a small portion of the population (Figure 3B). Moreover, GP82 colocalized with cruzipain later in the differentiation process, when parasites isolated from culture supernatant at 48 h were analyzed (Figure 3C-D). These results suggest that GP82 accumulates in LROs of intermediate forms, which are then directed to the flagellar pocket during differentiation to metacyclic forms, delivering GP82 to the plasma membrane.

## Discussion

Metacyclogenesis is a process whereby *T. cruzi* transforms from noninfective epimastigotes into infective metacyclic trypomastigotes and comprises a progressive morphological transformation, including transitional forms described as intermediate [13]. In this study, we demonstrated that GP82 and GP90 transcript levels increase in parasites forms undergoing differentiation and this increase is accompanied by translation of GP82 and GP90 proteins in intermediate forms. Several studies have shown that GP82 and GP90 are expressed by metacyclic trypomastigotes, but not by epimastigotes [18,19,21,22,41]. These results are in agreement with proteomic and transcriptomic analyzes of *T. cruzi* life-cycle stages, which revealed that GP82 and GP90 are up-regulated in metacyclic forms [42,43]. Despite those studies, little information was available about the expression of GP82 and GP90 during metacyclogenesis. In a previous work, different time points during metacyclogenesis were studied by proteomic analysis, revealing the presence of GP90 in parasites attached to culture flasks 24 h after nutritional stress [12]; however, this study was not able to detect GP82. In addition, although with no statistical significance, another quantitative proteomic study of *T. cruzi* metacyclogenesis identified different members of the *trans*-sialidase family expressed at higher levels in intermediate forms compared to epimastigotes [44].

In trypanosomes, gene expression is regulated at the posttranscriptional level mostly via mRNA stability and at least three known factors modulate mRNAs steady-state levels: *cis*-acting elements, *trans*-acting factors and the apparatus involved in mRNA turnover and degradation [45]. We have previously shown that GP82 transcripts accumulate in metacyclic forms and that treatment with translation inhibitors increased GP82 mRNA half-life in epimastigotes, suggesting that protein factors act by destabilizing transcripts in the epimastigote stage [22,46]. Thus, our findings support the notion that *trans*-acting factors that regulate GP82 and GP90 mRNAs steady-state levels are already operating in intermediate forms. Furthermore, it has been reported that transcriptional activity is constant during metacyclogenesis, but it is reduced in fully differentiated metacyclic trypomastigotes, when RNA Pol II disassembles from the nucleus [13]. Distinct from epimastigotes that replicate within the insect vector, metacyclic trypomastigotes are non-dividing forms that rapidly differentiate into amastigotes upon transmission to mammalian host, so it makes sense that these forms present a reduced transcriptional activity. Thus, we speculate that the majority of GP82 and GP90 mRNAs are transcribed and stabilized in intermediated forms and then accumulate in metacyclic forms.

The mechanisms involved in exocytosis, endocytosis and recycling in *T. cruzi* are still poorly understood compared to mammalian cells or to the closely related organism *Trypanosoma brucei*[47,48]. Most of what is known comes from structural and biochemical studies focused on morphology and localization of enzymes and endocytic markers [38-40,49-52]. Attempts to characterize and localize protein markers of intracellular compartments, e.g. Rab5, Rab7, Rab11 and Rho1, were unsuccessful and/or not fully explored [53-56]. We found that during nutritional stress GP82 and GP90 accumulate in distinct compartments in intermediate forms, despite their localization on the cell surface of metacyclic forms, indicating the existence of different control mechanisms to access the cell surface. The basis for such a difference is unclear, but it may be related to specific differences in the primary amino acid sequence and/or to posttranslational modifications, for instance, in the GPI anchor [57] and/or other lipid modifications [58]. Further studies will be needed to identify the mechanism responsible for delivering GP82 into LROs of intermediate forms and for the difference in protein localization observed between intermediate and metacyclic forms.

An increasing number of proteins with varied functions have being identified to localize in LROs, such as 52-kDa protein homologue to glutathione-S-transferase (Tc52) [59], farnesylated protein tyrosine phosphatase [60], β-tubulin [61], and RNA-binding protein (TcRBP40) [62]. In addition, subcellular proteomic analysis of reservosomes (LROs) fractions revealed a great variety of proteins with different functions; among them were surface proteins such as protease GP63, dispersed gene family protein-1, procyclic surface glycoprotein and kinetoplast membrane protein-11 [63]. Cunha-e-Silva *et al*. [64] suggested that LROs could be a heterogeneous population of organelles with different purposes, presenting storing, recycling and lysosome functions according to their maturation state. Our results are in agreement with this multipurpose hypothesis and also support the notion that the parasite nutritional state and developmental form influence the function of LROs. As endocytosis is very low or absent in metacyclic trypomastigotes compared to epimastigotes [65], our results suggest that LROs play a different role during metacyclogenesis and in metacyclic trypomastigotes compared to epimastigotes. Further characterization of the LROs population will help to clarify these findings.

The finding that GP82 colocalizes with cruzipain in LROs of intermediate forms, and that both proteins are directed to the plasma membrane with metacyclogenesis progression, may be relevant for the infective properties of metacyclic trypomastigotes. In preliminary experiments, we have found that cruzipain is involved in metacyclic trypomastigotes host cell invasion through a mechanism distinct from that described for tissue culture trypomastigotes (TCT) (unpublished data). Therefore, we suppose that upon GP82-mediated binding to target cells, cruzipain contributes for effective parasite internalization through a mechanism as yet to be elucidated.

## Conclusions

**Figure 4:**
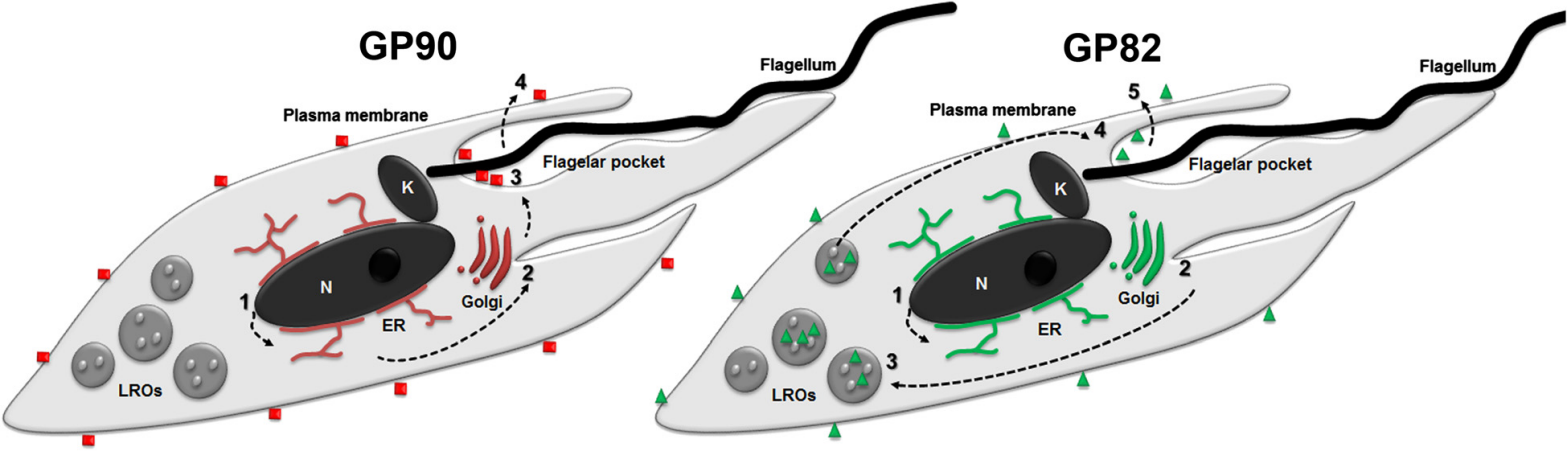
**Schematic representation of GP82 and GP90 pathways toward the cell surface during differentiation. **Intermediate forms in which these proteins start to be expressed are represented. GP90 (red squares) mRNAs sequences encode an N-terminal signal peptide and a C-terminal signal anchor driving polypeptide through the ER-Golgi secretory pathway (**1-2**) to be glycosylated and receive GPI anchor. GP90 proteins leave the Golgi complex in secretory vesicles that fuse with the flagellar pocket membrane (**3**) and are distributed along the parasite plasma membrane (**4**). GP82 (green triangles) mRNAs also encode N-terminal and C-terminal signal sequences that drive polypeptide through the ER-Golgi pathway (**1-2**) to receive post-translational modifications (glycosylation and GPI anchor). However, instead of being addressed straight to the plasma membrane via flagellar pocket, GP82 leave the Golgi complex in vesicles that concentrate in lysosome-related organelles (LROs) (**3**). Further in the differentiation process, sorting mechanisms occurring at LROs and organelle repositioning allow vesicles carrying GP82 to fuse with the flagellar pocket membrane (**4**) and distribute the protein along the plasma membrane (**5**). N: nucleus, K: kinetoplast.

This study discloses new aspects of protein expression and trafficking during *T. cruzi* differentiation by showing that the machinery involved in GP82 and GP90 gene expression starts to operate early in the differentiation process and that different secretion pathways are responsible for delivering these glycoproteins toward the cell surface (Figure 4).

## Competing interests

The authors declare that they have no competing interests.

## Authors’ contributions

The authors EBS and JFS idealized the project, contributed to the experimental design and wrote the manuscript. EBS performed the experiments and analyzed the data. NLCS and NY helped with discussions and manuscript elaboration. All authors read and approved the final version of the manuscript.

## Supplementary Material

Additional file 1 **Flow cytometry analysis of parasite populations undergoing differentiation. **Histograms showing there is a shift in fluorescence intensity in the whole parasite population obtained at 24 h and 48 h compared with epimastigote sample, indicating that intermediate forms are responsible for the signal and not the 2-3% metacyclic forms contamination. Click here for file
