# Supplementary material for: Expression and cellular trafficking of GP82 and GP90 glycoproteins during Trypanosoma cruzi metacyclogenesis
Source: Parasit Vectors. 2013 May 1;6:127. doi: 10.1186/1756-3305-6-127 (PMC3652755; doi:10.1186/1756-3305-6-127)
Supplement: Additional file 1 — Flow cytometry analysis of parasite populations undergoing differentiation. Histograms showing there is a shift in fluorescence intensity in the whole parasite population obtained at 24 h and 48 h compared with epimastigote sample, indicating that intermediate forms are responsible for the signal and not the 2-3% metacyclic forms contamination. [file 1756-3305-6-127-S1.pdf]

Additional file 1

Flow cytometry analysis of parasites populations

GP82

GP90

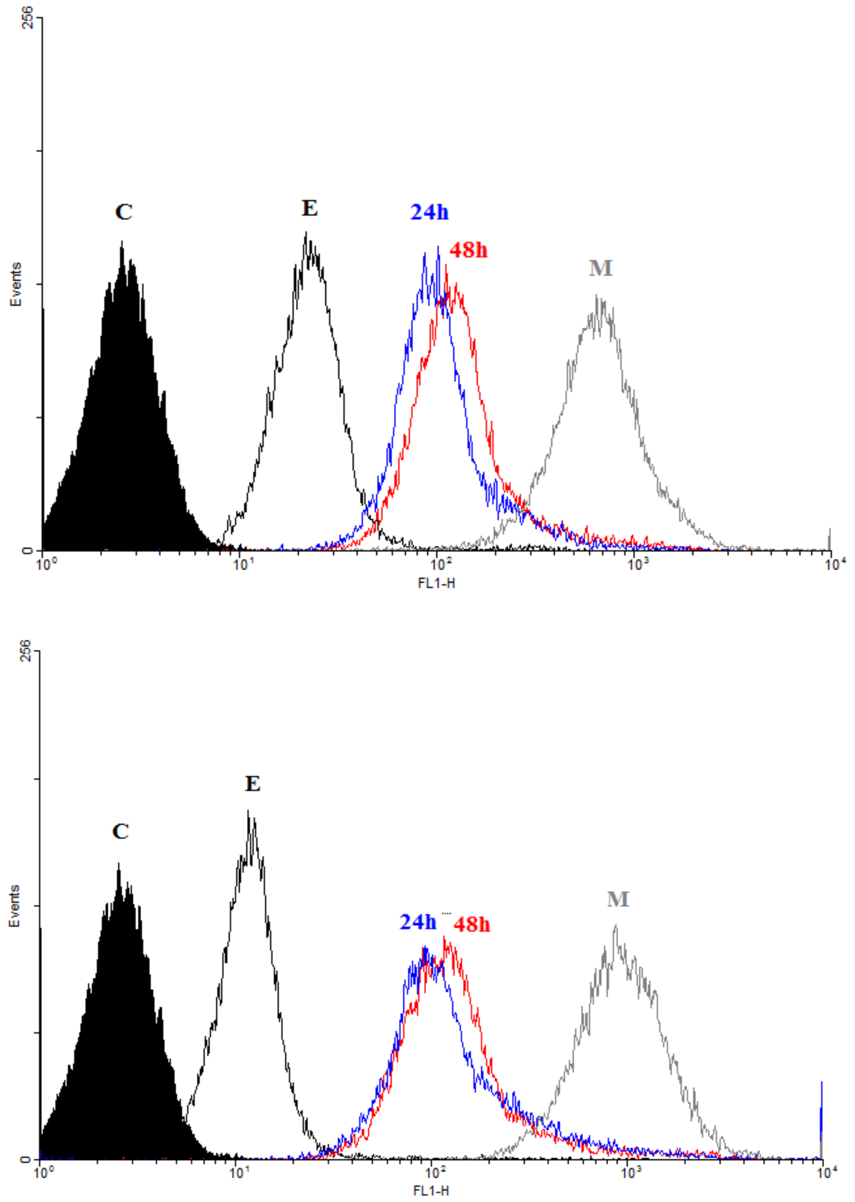

Legend:
Control (C): epimastigotes incubated with Alexa Fluor 488
Epimastigotes (E): epimastigotes incubated with mAb 3F6 or 1G7 followed by Alexa Fluor 488
Attached parasites 24 h (24h): intermediate forms incubated with mAb 3F6 or 1G7 followed by Alexa Fluor 488
Attached parasites 48 h (48h): intermediate forms incubated with mAb 3F6 or 1G7 followed by Alexa Fluor 488
Metacyclic forms (M): metacyclic forms incubated with mAb 3F6 or 1G7 followed by Alexa Fluor 488
